# Influence of the Madden–Julian oscillation on Tibetan Plateau snow cover at the intraseasonal time-scale

**DOI:** 10.1038/srep30456

**Published:** 2016-07-28

**Authors:** Wenkai Li, Weidong Guo, Pang-chi Hsu, Yongkang Xue

**Affiliations:** 1 Institute for Climate and Global Change Research, School of Atmospheric Sciences, Nanjing University, Nanjing, China; 2 Joint International Research Laboratory of Atmospheric and Earth System Sciences, Nanjing, China; 3 Key Laboratory of Meteorological Disaster of Ministry of Education/Joint International Research Laboratory of Climate and Environment Change/Collaborative Innovation Center on Forecast and Evaluation of Meteorological Disasters, Nanjing University of Information Science & Technology, Nanjing, China; 4 Department of Geography and Department of Atmospheric and Oceanic Sciences, University of California, Los Angeles, California, USA

## Abstract

The Tibetan Plateau (TP), known as the third pole of the Earth, has snow cover with intraseasonal to decadal variability that affects weather and climate both inside and outside the TP. However, the factors that generate the TP snow cover (TPSC) anomalies at the intraseasonal time-scale are unclear. This report reveals the influence of the Madden‒Julian oscillation (MJO), which is the most dominant component of the tropical intraseasonal variability, on TPSC. We focus on wintertime snow cover over the central and eastern TP, where the intraseasonal variability is large. TPSC increases/decreases in the MJO phases 8‒1/4–5, when the eastward-propagating MJO suppressed/enhanced convection locates over the Maritime Continent. Such a change in TPSC leads to the most dominant positive/negative anomalies of TPSC in the following phases 2‒3/6‒7 due to the non-significant change of TPSC in these phases. There is anomalous moisture advection over the upstream of the TP caused by MJO-excited large-scale atmospheric circulation. The advection process generates the low-frequency eastward-propagating anomalous water vapour from upstream to the TP that influences precipitation and, eventually, TPSC.

Snow cover is a crucial component in the cryosphere. It affects the global climate system^1^ through the sensitivity of the radiation balance to the high albedo characteristics of snow^2^,^3^,^4^,^5^ and the energy allocation involved in the melting of snowpack^6^,^7^. The Tibetan Plateau (TP), which is the largest and highest plateau in the world and is known as the third pole^8^, has snow cover with multiple time-scale (intraseasonal to decadal) variability^9^ that affects both weather and climate. The variability of TP snow cover (TPSC) on the interannual to decadal time-scales has been extensively investigated^5^,^10^,^11^,^12^,^13^,^14^,^15^,^16^. However, the factors that generate TPSC anomalies at the intraseasonal time-scale are unclear. A better understanding of the intraseasonal variability of TPSC is valuable for comprehending the atmospheric activity over the TP at the intraseasonal time-scale, which exerts influences both inside and outside the TP^17^,^18^,^19^. Furthermore, the TP is the source of many major rivers in Asia. A large part of the precipitation in the upper streams of these rivers on the TP falls in the form of snow, thus causing a natural delay of river discharge^12^. Understanding the factors that govern the intraseasonal variability of TPSC is also critical for water management in downstream regions.

The Madden–Julian oscillation^20^,^21^ (MJO) is the most dominant component of the tropical intraseasonal variability. The MJO has planetary-scale signals in atmospheric circulation systems coupled with deep convection and propagates eastwards slowly (~5 m/s) from the Indian Ocean to the Pacific Ocean^22^. During its eastward propagation, the circulation anomalies associated with the MJO interact with various weather and climate systems^23^, affecting precipitation^24^,^25^,^26^,^27^,^28^,^29^,^30^, monsoons^31^,^32^,^33^, temperature^28^,^34^, tropical cyclones^35^,^36^, El Niño–Southern Oscillation^37^,^38^,^39^, polar circulations^40^,^41^,^42^, Arctic sea ice^43^ and snowpack^44^,^45^. However, compared to other weather and climate systems, few studies have focused on the MJO-related intraseasonal variability of snow cover on the TP. As the MJO becomes increasingly predictable^46^, understanding the influence of the MJO on all aspects of the Earth’s system, including snow cover on the TP, allows for a better prediction of snow cover at an extended-range (10–30 days).

Motivated by the above reasons, this work aims to investigate that the influence of the MJO on TPSC.

## Results

### Signatures of the eastward propagating MJO convection

**Figure 1:**
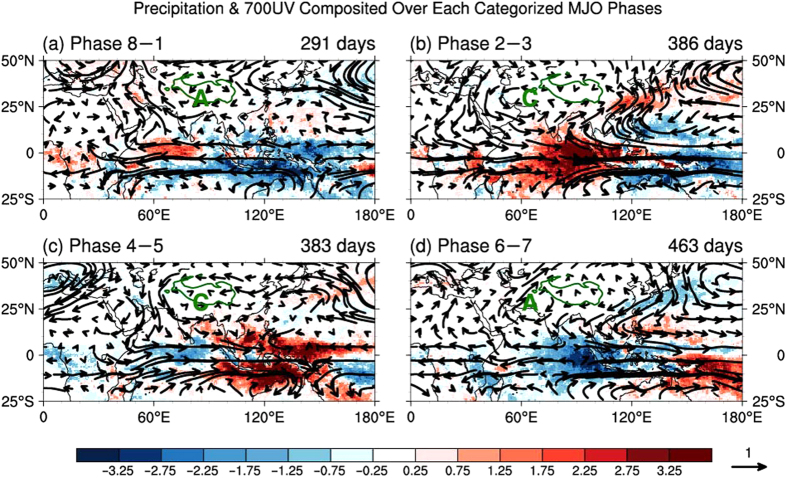
Signatures of the eastward propagating MJO convection and corresponding responses of low-level large-scale atmospheric circulations. Wintertime composites of the 20–100-day filtered daily anomalous precipitation (shading; unit: mm day^−1^) and 700 hPa winds (vectors; unit: m s^−1^) are shown for the categorized phases. Red colours correspond to enhanced convective activity and diabatic heating, and blue colours correspond to reduced convective activity and diabatic cooling. The reference magnitude used for the winds is 1 m s^−1^. The green contour outline marks the Tibetan Plateau. The interval of colour bar is 0.5 mm day^−1^. The “A” and “C” stand for anticyclone and cyclone, respectively. Figure 1 was generated using NCAR Command Language (NCL) version 6.3.0, an open-source software package that is free to the public and was developed by UCAR/NCAR/CISL/TDD, http://dx.doi.org/10.5065/D6WD3XH5.

In this article, the MJO states are tracked by the daily all-season real-time multivariate MJO (RMM) index proposed by Wheeler and Hendon^47^. The RMM index has been widely applied to diagnose the influence of the MJO on various weather and climate systems^25^,^26^,^29^,^30^,^34^,^36^,^40^,^41^,^42^,^43^,^44^,^45^. The RMM index describes a MJO cycle that generally progresses eastward from phase 1 to phase 8. The nominal time for the transition between each of the numbered phases is 6 days but can vary from event to event. To make the results more concise, we categorized the eight MJO phases into four groups (i.e., Phase 8–1, Phase 2–3, Phase 4–5 and Phase 6–7) following Ref. 26. The eastward propagating convection is one of the most basic features of the MJO^22^. Here, we describe convection by using precipitation data. Wintertime (Nov.‒Mar.) composites of the 20–100-day filtered daily anomalous precipitation for the four categorized phases of the MJO (shading in Fig. 1) depict the eastward-propagating MJO convection. Positive/negative precipitation anomalies correspond to enhanced/suppressed convective activity. The composite in phase 2‒3 shows a dipole structure of convection anomalies (Fig. 1b). Enhanced convection is located in the eastern Indian Ocean, whereas reduced convection is located in the western Pacific. In the subsequent phase 4‒5 (Fig. 1c), the enhanced convective anomaly moves eastwards, positioning over the Maritime Continent (90‒150°E). Then, in phase 6–7 (Fig. 1d), the convection moves into the western Pacific, and suppressed convection develops over the eastern Indian Ocean. In phase 8–1 (Fig. 1a), the suppressed convection moves into the Maritime Continent and returns to phase 2–3. The anomalous convection in phase 2–3 is opposite to that found in phase 6–7. Further, the composites in phases 4–5 and 8–1 are also out of phase. In the next section, we present the response of TPSC to this eastward-propagating MJO convection.

### Relationships between the MJO and Tibetan Plateau snow cover

This study spans 16 extended winters (from Nov. 1, 1998 to Mar. 31, 2014) and is focused on the intraseasonal variability of the daily TPSC. Each winter comprises 151 days from Nov. 1 to Mar. 31 of the next year. Most areas of the central and eastern TP are covered by snow for approximately 10–30% of wintertime (Supplementary Fig. S1a), which is much less than that of the western TP, where snow covers the ground for more than 80% of the time. The central and eastern parts of the TP have the largest intraseasonal (20–100-day) variabilities (Supplementary Fig. S1b), whereas the variability is much smaller in the western TP. Therefore, the TP in this study is defined as the area with an altitude greater than 3,000 meters and within 27–40°N and 80–105°E covering the central and eastern parts of the TP, where the main modulation of the MJO on TPSC occurs.

**Figure 2:**
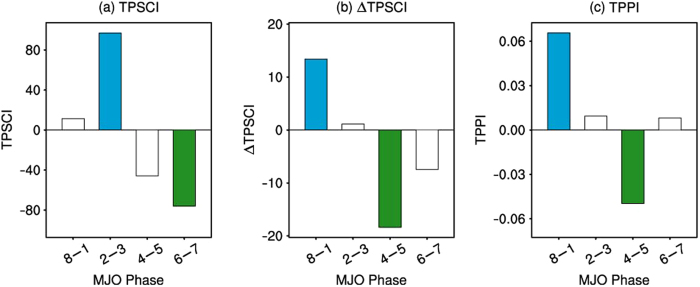
Relationships between the categorized phases of the eastward propagating MJO and the indices of snow cover, daily change of snow cover and precipitation over the Tibetan Plateau. Wintertime composites of the 20–100-day filtered daily anomalous (**a**) Tibetan Plateau Snow Cover Index (TPSCI; unit: number of grid points); (**b**) daily change of the TPSCI (ΔTPSCI; unit: number of grid points); (**c**) Tibetan Plateau Precipitation Index (TPPI; unit: mm day^−1^) for the categorized MJO phases. The definition of the three indices can be found in the Methods. The coloured bars indicate the composites that are significant at the 99% confidence level based on the Monte Carlo test, and the white bars indicate non-significant composites. For the blue and green bars, the indices are significantly positive or negative, respectively. Figure 2 was generated using NCAR Command Language (NCL) version 6.3.0, an open-source software package that is free to the public and was developed by UCAR/NCAR/CISL/TDD, http://dx.doi.org/10.5065/D6WD3XH5.

**Figure 3:**
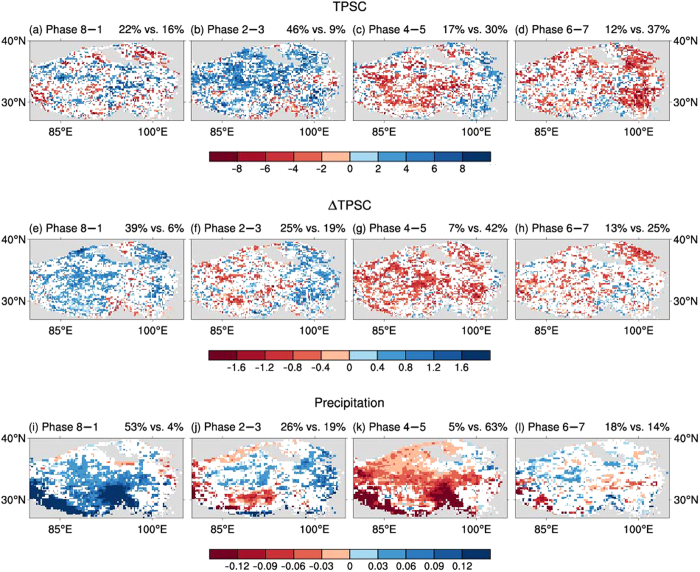
Relationships between the categorized phases of the eastward propagating MJO and snow cover, daily change of snow cover and precipitation over the Tibetan Plateau. (**a**–**d**) Wintertime composites of the 20–100-day filtered daily anomalous Tibetan Plateau snow-covered probability (unit: %). (**e**–**h**), as (**a**–**d**), but for daily change of snow cover. (**i**–**l**), as (**a**–**d**), but for precipitation (unit: mm day^−1^). Coloured grid points indicate the composites are significant at the 95% confidence level based on the Monte Carlo test, and the white grid points indicate non-significant composites. Grey areas outline the area with altitudes less than 3,000 meters. The right title for each figure represents the percentage of grid points with significant positive values vs. that with significant negative values. Figure 3 was generated using NCAR Command Language (NCL) version 6.3.0, an open-source software package that is free to the public and was developed by UCAR/NCAR/CISL/TDD, http://dx.doi.org/10.5065/D6WD3XH5.

To represent the TPSC regional variability, a TPSC index (TPSCI) is defined (see the Methods section for more details), which represents the number of snow-covered grid points over the TP in the analysis. Composites of the TPSCI for the categorized MJO phases are obtained by using the RMM index to examine the relationship between the MJO and the TPSC regional variability (Fig. 2a). The index shows a clear variation associated with the MJO. There is a positive composite of the TPSCI in phase 2–3 that is significant at the 99% confidence level. In contrast, in phase 6–7, there is a negative composite of the TPSCI that is significant at the 99% confidence level. There is a dramatic switch from phase 2–3 to phase 6‒7, and the signatures of MJO convection are out of phase, as revealed in Fig. 1. However, the composited anomalies are not significant in phases 8–1 and 4–5. The magnitude of the composites of the standardized anomalies of the TPSCI in phase 2–3 is 0.23, whereas in phase 6–7, it is ‒0.14, which represents approximately 20% of its standardized variability on the intraseasonal time-scale. We also show the spatial distribution of the composites for each grid point over the TP (Fig. 3a‒d). In phase 2‒3 (Fig. 3b), significant positive anomalies are found in 46% of the grid points over the TP, which corresponds to increased snow-covered probability. In phase 4‒5 (Fig. 3c), negative anomalies occur over the central TP, but positive anomalies are found over some area of the eastern TP. In phase 6‒7 (Fig. 3d), negative anomalies are found over 37% of the grid points, which corresponds to decreased snow-cover probability. In phase 8‒1 (Fig. 3a), the spatial patterns of positive and negative anomalies are quite mixed, with no dominant patterns observed. Both the composites of the TPSCI and the composites for each grid point suggest that the most dominant positive/negative anomalies of the overall TPSC occur in the MJO phases 2‒3/6‒7.

To further investigate the change of snow cover, we analysed the relationship between the daily change of TPSC (ΔTPSC) and the MJO. The daily change of the TPSCI (ΔTPSCI) is the difference between the TPSCI for one day and the TPSCI for the previous day (see the Methods section for more details). A positive/negative ΔTPSCI corresponds to an/a increasing/deceasing TPSCI. Composites of the ΔTPSCI for the categorized MJO phases are examined (Fig. 2b). The ΔTPSCI also shows a clear MJO-related variation. There is a positive composite of the ΔTPSCI in phase 8–1 that is significant at the 99% confidence level. In contrast, in phase 4–5, there is a negative composite of the ΔTPSCI that is significant at the 99% confidence level. Namely, the TPSCI significantly increases in phase 8–1 but decreases in phase 4–5. However, the composites in phases 2–3 and 6–7 are non-significant. The composites of ΔTPSC for each grid point (similar to the ΔTPSCI, but for each grid point) are also investigated (Fig. 3e‒h). In phase 8‒1 (Fig. 3e), a significant and positive anomalous ΔTPSC is found in 39% of the total grid points over the TP, whereas the significant and positive anomalous ΔTPSC is only found in 6% of the grid points. In contrast, negative anomalies are found over most of the anomalous grid points, with significance in phase 4‒5 (Fig. 3g). In phases 2‒3 and 6‒7 (Fig. 3f,h), the spatial patterns of positive and negative anomalous ΔTPSC are mixed, without uniform negative or positive anomalies. The spatial distributions of the composites for ΔTPSC are consistent with the composites of the ΔTPSCI. Both the composites of the ΔTPSCI and the composites of ΔTPSC for each grid point suggest that TPSC increases/decreases in the MJO phases 8‒1/4‒5.

The change of TPSC has an inherent relationship with the anomalous TPSC. In phase 8–1, the ΔTPSCI is positive, which means that the TPSCI increases in this phase. When phase 8–1 ends and the following phase 2–3 starts, the TPSCI reaches its peak value. Due to the non-significant anomalous ΔTPSCI in phase 2–3, the TPSCI keeps the positive peak value in this phase. For a similar reason, the negative anomalous ΔTPSCI in phase 4–5 leads to the negative peak value of the TPSCI in the consequent phase 6–7. The TPSCI lags that of ΔTPSCI by two phases of a MJO cycle (1/4 cycle). A theoretical explain on this relationship can be referred in the Method section. Such a relationship is also generally applicable for the spatial grid points.

The above analysis reveals that TPSC increases/decreases in the MJO phase 8‒1/4–5, when the eastward-propagating MJO suppressed/enhanced convection is located over the Maritime Continent. Such a change of TPSC leads to the positive/negative peak anomalies of TPSC in their following phase 2‒3/6‒7 due to the non-significant change of TPSC in these phases. We also get similar results based on regression method, which can be referred in the Supplementary Information (Fig. S2a–b). To further understand this MJO phase and TPSC relationship, in the next section, we discuss the relationship between the MJO and TP precipitation.

### Relationship between the MJO and precipitation over the Tibetan Plateau

Due to the very cold temperature over the wintertime TP, wintertime precipitation occurs mainly in the form of snow. The anomalous precipitation contributes to the anomalies of snow cover where the surface temperature is below the freezing point^11^,^44^. Therefore, precipitation plays a key role in the variability of the TPSC. Previous studies have found that the influence of the MJO on rainfall is significant almost globally^25^, including the rainfall variability in East Asia^26^,^28^,^29^,^30^. However, to date, few works have focused on the influence of the MJO on precipitation over the TP. As such, in this study, we examine the MJO’s influence on precipitation over the TP.

We first calculated the TP Precipitation Index (TPPI) by averaging the precipitation at each grid point over the TP. Composites were then calculated for the 20‒100-day filtered anomalous TPPI (Fig. 2c). We found that there are significant influences of the MJO on precipitation over the TP. There is more precipitation in phase 8‒1 but less precipitation in phase 4‒5, and these composites are significant at the 99% confidence level. However, the anomalous TPPI is non-significant in phases 2–3 and 6–7. We also get consistent results based on regression method, which can be referred in the supplementary (Fig. S2c). The spatial distributions of composites of precipitation show similar results (Fig. 3i‒l). Positive anomalies are found over most anomalous grid points with significance in phase 8‒1 (Fig. 3i), and the significant negative anomalies occur in phase 4‒5 (Fig. 3k). In other phases, the spatial patterns of precipitation show fewer anomalies (Fig. 3j,l). The spatial distributions of the composites for precipitation are consistent with the composites of the TPPI. Both the composites of the TPPI and the composites for precipitation over each grid point suggest that the precipitation over the TP increases/decreases significantly in the MJO phases 8‒1/4‒5.

It is not a surprise that the relationship between the TPPI and the MJO is the same as that between the ΔTPSCI and the MJO (Fig. 2b‒c). The greater amount of precipitation that occurs in phase 8–1 is in favour of the increase of TPSC. Therefore, the ΔTPSCI is also significantly positive. Conversely, the lower levels of precipitation in phase 4‒5 lead to the negative ΔTPSCI in phase 4‒5. The non-significant anomalous TPPI in phases 2–3 and 6–7 corresponds to the non-significant anomalous ΔTPSCI in these phases. The composites for each grid over the TP show similar results. The increased precipitation over most grid points in phase 8‒1 (Fig. 3i) is generally consistent with the most positive anomalous ΔTPSC (Fig. 3e), except for some grid points over the eastern part of the Himalayas (near 30°N, 95°E), where the climatology of wintertime snow is different from that of the surrounding areas (Fig. S1). In phase 2–3, both composites of ΔTPSC and precipitation over the grid points (Fig. 3f,j) feature a dipole pattern with opposite signs of anomalies over the central and eastern TP. However, the number of grid points with significant anomalies is less than that observed in phases 8–1 and 6–7. The negative anomalies show eastwards propagation. In the following phase 4‒5, both composites of ΔTPSC and precipitation over the grid points show coincident negative anomalies (Fig. 3g,k). In phase 6‒7, the spatial patterns of both ΔTPSC and precipitation are quite mixed (Fig. 3h,l), and the number of grid points with significant anomalies is relatively low.

### Mechanism

To investigate the mechanism for the above modulation by the MJO, we examine the evolution of the large-scale atmospheric circulation anomalies associated with the MJO and their link to snow cover and precipitation over the TP. It is well known that tropical diabatic heating associated with the MJO excites subtropical planetary waves via barotropic vorticity perturbations which then propagate poleward^40^,^41^,^48^,^49^,^50^,^51^. Gill^48^ described the large-scale circulation that forms in response to prescribed localized steady heating associated with deep tropical convection. He used a linear damped shallow-water equation model on an equatorial plane that provided elegant analytical solutions for some particular heating distributions. In the Gill’s model, the feature of the tropical atmospheric response to the MJO diabatic heating is a pair of anomalous cyclones symmetric about the equator to the west of the heating. The cyclones are associated with an equatorial Rossby wave structure. The response can be found in real atmospheric circulation through composites to certain MJO phases according to our study. The eastward propagation of the anomalous convection pattern is associated with modifications of low-level atmospheric circulation (vectors in Fig. 1). When the major enhanced convection (corresponding to upward vertical motion) of the MJO moves into the eastern Indian Ocean (Phase 2–3, Fig. 1b), the diabatic heating of convection excites the classic Matsuno–Gill pattern^48^,^52^. The tropical atmospheric response to the MJO diabatic heating is characterized by a pair of low-level cyclones symmetric about the equator to the west of the diabatic heating region. The centre of the boreal cyclone is located near 25°N 60°E (marked by “C” in Fig. 1b). Such a feature is also found in previous studies^24^,^27^,^28^,^30^,^35^. The cyclone moves eastwards, following the eastward progression of the MJO convection. Due to the huge blocking effect of the TP, the features of a cyclone are not well developed in phase 4–5 (Fig. 1c). However, when the suppressed MJO convection (corresponding to downward vertical motion) moves from the western Indian Ocean to the Marine Continent (Phase 6–7 and 8–1, Fig. 1d,a), the anomalies are almost opposite to the anomalies induced by the enhanced MJO convection. There is an anticyclone moving eastwards (marked by “A” in Fig. 1d,a), following the movement of the suppressed MJO convection.

The wave perturbations related to the Matsuno–Gill-type pattern that induced by the MJO are able to influence northern hemispheric weather patterns^24^,^27^,^28^,^30^,^35^. Our studies found that the Matsuno–Gill-type response further influences the weather over the Tibetan Plateau. As the above mentioned, the Matsuno–Gill-type response features an anticyclone/cyclone in the northwest of the diabatic-cooling/heating region (marked by “A”/“C” in Fig. 1). Note that there are southerly/northerly winds in the western part of the anticyclone/cyclone. The southerly anomalies are located over the Arabian Peninsula in phase 6−7 and move eastwards in the following phase 8−1. In phase 8−1, the Arabian Sea, Arabian Peninsula and Iranian Plateau are controlled by the southerly wind anomalies. On the other hand, the northerly wind anomalies are located over the Arabian Peninsula in phase 2–3 and move eastwards in the following phase 4‒5. In phase 4‒5, the Arabian Sea, Arabian Peninsula and Iranian Plateau are controlled by these northerly wind anomalies. The southerly wind anomalies over the Arabian Sea may bring water vapour from the sea to the land and increase the moisture over the area upstream of the TP, whereas the northerly wind anomalies from the land to the sea reduce the moisture. To prove this hypothesis, we analysed the vertical integral of water vapour and its flux related to the activity of the MJO (Fig. S3). Because most moisture in the atmosphere exists in the low-level region, the vertical integral of water vapour flux shows a similar pattern to that of the horizontal wind at 700 hPa (Fig. 1). There is a southerly anomalous water vapour flux over the Arabian Sea, Arabian Peninsula and Iranian Plateau in phase 8‒1 (Fig. S3a), which is consistent with the low-level southerly wind anomalies over this area (Fig. 1a). The water vapour flux influences the amount of vapour in atmosphere. The southerly anomalous water vapour flux brings moisture from the Arabian Sea to the Arabian Peninsula and Iranian Plateau. As a result, a positive anomalous vertical integral of water vapour occurs over the Iranian Plateau (greater than 1.0 kg m^−2^). In contrast, the northerly anomalous water vapour flux over this area leads to an opposite result (Fig. S3c). The water vapour shows negative anomalies in phase 4‒5 over the Iranian Plateau (less than −1.0 kg m^−2^).

Nazemosadat and Ghaedamini^27^ found that the enhanced low-level southerly winds excited by the suppressed MJO convection over the Maritime Continent transfer a substantial amount of moisture to Southern Iran and the Arabian Peninsula. Precipitation over these areas is then influenced. Our study is consisting with their work. And we further found that the anomalous water vapour propagates eastwards and reaches the TP due to an advection process. In the wintertime, low-level westerly winds occur over the area upstream of the TP. The low-level westerly winds that climb up the TP bring water vapour from the west of the TP to the central and eastern TP, thereby forming a moisture channel. The anomalous water vapour occurs over the area upstream of the TP (Iranian Plateau) and influences the moisture over the TP through the advection caused by the background of westerly winds. Hovmöller diagrams, averaged between 27° and 40°N, illustrate the zonal propagation characteristics of the low-frequency water vapour from the area upstream of the TP (Figs S4 and S5). Figure S4 shows the zonal propagation of 20‒100-day filtered anomalous vertical integral moisture horizontal advection. The anomalies of moisture horizontal advection *Q*_*adv*_′ is calculated by


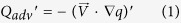


where 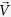 represents horizontal winds, *q* represents specific humidity, 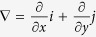, a prime denote the intraseasonal component (20–100-day). The intraseasonal component is extracted by applying band-pass filter. Then, the moisture horizontal advection at each pressure level is integrated from the surface pressure to 100 hPa. The 20–100-day filtered anomalies *Q*_*adv*_′ is calculated by using the same method as Hsu and Li^53^ used. The moisture horizontal advection represents the process of transport of moisture by the mass motion (horizontal velocity field) of the atmosphere. A positive/negative moisture advection means that horizontal winds contribute to increasing/decreasing moisture at a local point. There is positive anomalous moisture advection starting from 70°E on day −8 at phase 8–1 (Fig. S4a). This anomaly propagates eastwards and reaches the TP (80‒105°E) at phase 8‒1. The positive anomalous moisture advection will increase the water vapour over the TP due to horizontal wind transportation. It can be see that positive anomalous water vapour propagates eastwards and reaches the TP in phase 8‒1 (Fig. S5a). The positive anomalous water vapour supports precipitation. Therefore, there is positive anomalous precipitation characterized by eastward propagation (Fig. S6a). The anomalous eastward-propagating precipitation reaches the TP in phase 8‒1. The situation is opposite in phase 4‒5. There is negative anomalous moisture advection with eastward propagation (Fig. S4c), and the moisture decreases (Fig. S5c). As a result, the negative anomalous precipitation propagates eastwards and reaches the TP in phase 4‒5 (Fig. S6c). For phases 2‒3 and 6‒7, the interval between the eastward-propagating low-frequency negative and positive moisture advection and water vapour anomalies reaches the TP (Fig. S4b and S4c; Fig. S5b and S5c). Therefore, the precipitation over the TP does not show significant anomalies in phases 2‒3 or 6‒7 (Fig. S6b and S6c). The mechanism is summarized in Fig. S7.

## Summary and Discussion

This report presents the influence of the MJO on TPSC. We focus on wintertime snow cover over the central and eastern parts of the TP at an altitude greater than 3,000 meters, where the intraseasonal variability is large. The TPSC increases/decreases in the MJO phases 8‒1/4–5, when the eastward-propagating MJO suppressed/enhanced convection is located over the Maritime Continent. Such a change of TPSC leads to the most dominant positive/negative anomalies of TPSC in the following phases 2‒3/6‒7 due to the non-significant change of TPSC in these phases.

We further investigated the modulation of the MJO on precipitation over the TP, which contributes to the change of TPSC. The diabatic heating of MJO-suppressed/enhanced convection excites the classic Matsuno–Gill pattern, which features an anticyclone/cyclone in the northwest of the diabatic-cooling/heating region. The southerly/northerly wind anomalies associated with the anticyclone/cyclone over the Arabian Sea increase/decrease the moisture over the area upstream of the TP. The anomalous water vapour propagates eastwards and reaches the TP due to an advection process, thus causing positive/negative anomalies of water vapour over the TP. The precipitation over the TP is subsequently influenced, and TPSC is eventually affected. The mechanism of this modulation is summarized in Figure S7.

This report provides a first look at the influence of the MJO on TPSC and suggests a possible mechanism for the modulation of the MJO on TPSC. However, further work is needed, including numerical experiments. Because TPSC significantly affects albedo, it may play an important role in linking the MJO and the meteorological and hydrologic elements over the TP, such as the surface energy budgets. More related work in the future is necessary for a better understanding of the MJO modulation on weather and climate in the TP. Although the MJO is the most dominant component of the tropical intraseasonal variability, the intraseasonal variability of weather can also be influenced by other potential factors. For example, the oscillation with higher frequency (10–30-day)^19^,^54^ and subtropical jet^18^ may be related the variability of Tibetan Plateau snow cover. More detail work on this topic will be in our future study.

## Methods

### Data

Four publicly available datasets are used in this study.

1. The daily all-season real-time multivariate MJO (RMM) index proposed by Wheeler and Hendon^47^ was used as a measure of the MJO states. The daily RMM data are available online at http://www.cawcr.gov.au/staff/mwheeler/maproom/RMM/. The RMM index represents the propagating feature of the MJO from the tropical Indian Ocean to the western Pacific Ocean and divides the full cycle of the MJO into eight phases. The RMM index describes an MJO cycle that generally progresses eastwards, from phase 1 to 8 and back to phase 1 again. The amplitude of the MJO was defined as 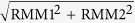. See Ref. 47 for more details about the RMM.

2. The daily snow cover data at 24-km resolution were obtained from the Interactive Multi-sensor Snow and Ice Mapping System (IMS) snow cover analysis^55^. The raw snow cover analysis data are binary; 1 indicates the grid point covered by snow, and 0 means non-snow-covered.

3. The precipitation dataset used is the Tropical Rainfall Measuring Mission (TRMM) daily precipitation (3B42)^56^.

4. Daily-averaged large-scale atmospheric circulation was obtained from ERA-Interim^57^. The horizontal winds at 700-hPa, the vertical integral of water vapour and its flux are directly obtained from this dataset. The vertical integral moisture advection is calculated by using horizontal winds and specific humidity at 10 pressure levels (1000, 925, 850, 700, 600, 500, 400, 300, 200 and 100 hPa).

### Filter

The annual cycle has been removed from all of the data used in this study to obtain anomalies. Because TPSC has multiple time-scale variability^9^, including interannual to decadal variability, a bandpass filter that isolates the 20–100-day components is applied for all of the variables discussed in this studies, except for climatology in Supplementary Figure S1a. For the snow cover of each grid point (Fig. 3), we also applied the bandpass filter to single out the intraseasonal component. When one grid point is covered by snow, we used a value of 100% to represent the probability of being snow-covered, whereas 0% indicates the non-snow covered points. The filtered values of snow cover data over each grid point indicate the probability of snow-cover at an intraseasonal time-scale.

### Composite

This study investigates the influence of the MJO through a composite analysis. The phase and amplitude of the MJO were defined using the daily RMM index. The RMM divides the state of the MJO into eight phases. Each day corresponds to a certain MJO phase. To make the results concise, we categorized eight MJO phases into four groups (i.e., Phase 8–1, Phase 2–3, Phase 4–5 and Phase 6–7) following Ref. 26. According to the RMM index, the elements of snow cover and atmosphere are divided into four categories. The composites are then derived by averaging each category. Here, only strong MJOs 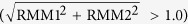 were contained for the composite, and weak MJOs were excluded. The number of days for each phase of the MJO is shown in the upper right corner of each panel in Fig. 1, and they are the sample size for the composite. This compositing methodology has been used in many previous studies^25^,^26^,^29^,^30^,^34^,^36^,^40^,^41^,^42^,^43^,^44^,^45^.

### Statistically significant test

The statistically significant test is based on Monte Carlo simulation. For each quantity, the difference between eight MJO phases and weak MJOs is tested for statistical significance, as follows. First, all of the 16-year wintertime (Nov.–Mar.) MJOs are pooled, and random resampling is used to create 1,000 samples. Then, the composite is calculated between eight MJO phases and weak MJOs; for each MJO phase and each sample, an empirical distribution of each quantity is produced. The observed value of each quantity is then compared to that at the 2.5^th^ (0.5^th^) and 97.5^th^ (99.5^th^) percentiles based on the empirical distribution. If the observed value is beyond the 2.5^th^ (0.5^th^) and 97.5^th^ (99.5^th^) percentiles, then the composites are considered statistically significant at the 95% (99%) confidence level; otherwise, they are non-significant.

### The definitions of TPSCI, ΔTPPI and TPPI

The Tibetan Plateau Snow Cover Index (TPSCI) is defined as


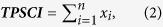


where x is the IMS snow cover analysis over the TP. If one grid point is covered by snow, x = 1; otherwise, x = 0. In other words, the TPSCI is the number of snow-covered grid points over the TP. The grid points over the TP are defined as points at an altitude greater than 3,000 meters and within 27–40°N and 80–105°E. In total, there are 6010 grid points over the TP according to this criterion.

The daily change of the TPSCI (**ΔTPSCI**) is the difference between the TPSCI for one day and the TPSCI for the previous day. The daily change of the TPSCI (ΔTPSCI) was calculated as


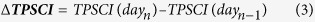


where TPSCI(day_*n*_) is the TPSCI for day *n* and TPSCI(day_n-1_) is the TPSCI for the previous day. Here the ΔTPSCI is actually the derivative of TPSC with respect to time, and is estimated by backward differentiation. If we express


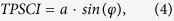


φ is the phase angle of the MJO. Then the


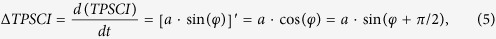


Therefore the TPSCI lags that of ΔTPSCI by 1/4 cycle.

The Tibetan Plateau Precipitation Index (**TPPI**) is defined the same as the TPSCI, except the TRMM precipitation is used, and the unit is mm day^‒1^.

### Graphic software

All figures were produced using NCAR Command Language (NCL)^58^.

## Additional Information

**How to cite this article**: Li, W. *et al.* Influence of the Madden-Julian oscillation on Tibetan Plateau snow cover at the intraseasonal time-scale. *Sci. Rep.* **6**, 30456; doi: 10.1038/srep30456 (2016).

## Supplementary Material

Supplementary Information
