# Supplementary material for: Influence of the Madden–Julian oscillation on Tibetan Plateau snow cover at the intraseasonal time-scale
Source: Sci Rep. 2016 Jul 28;6:30456. doi: 10.1038/srep30456 (PMC4964337; doi:10.1038/srep30456)
Supplement: Supplementary Information [file srep30456-s1.pdf]

Supplementary Information to

Influence of the Madden–Julian oscillation on Tibetan Plateau snow cover at the intraseasonal time-scale

Wenkai Li[1,2], Weidong Guo[1,2], Pang-chi Hsu[3] & Yongkang Xue[4]

1 Institute for Climate and Global Change Research, School of Atmospheric Sciences, Nanjing University, Nanjing, China.

2 Joint International Research Laboratory of Atmospheric and Earth System Sciences, Nanjing, China.

3 Key Laboratory of Meteorological Disaster of Ministry of Education/Joint International Research Laboratory of Climate and Environment Change/Collaborative Innovation Center on Forecast and Evaluation of Meteorological Disasters, Nanjing University of Information Science & Technology, Nanjing, China.

4 Department of Geography and Department of Atmospheric and Oceanic Sciences, University of California, Los Angeles, California, USA.

Correspondence and requests for materials should be addressed to W.G. (email: guowd@nju.edu.cn)

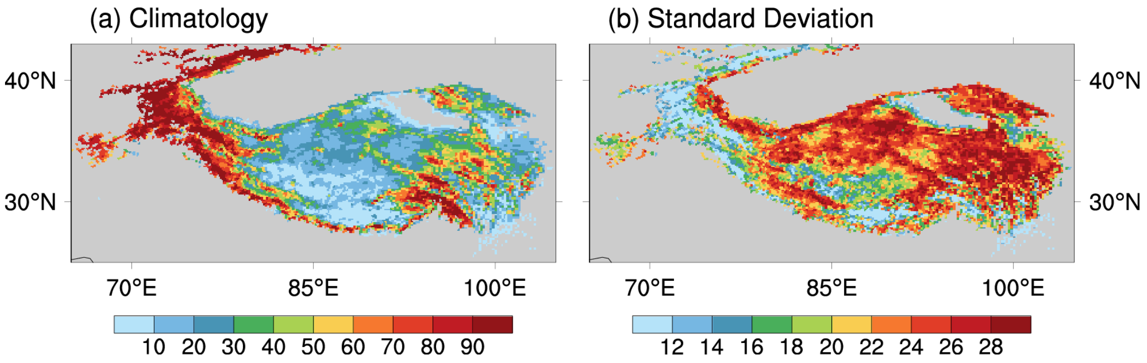

Figure S1. Climatological mean of wintertime Tibetan Plateau snow cover and its intraseasonal standard deviation. (a) The climatological probability of snow-cover occurrence over the Tibetan Plateau (Nov.–Mar.). (b) The stand deviation of the 20–100-day filtered anomalous probability. Unit is %. Figure S1 was generated using NCAR Command Language (NCL) version 6.3.0, an open-source software package that is free to the public and was developed by UCAR/NCAR/CISL/TDD, http://dx.doi.org/10.5065/D6WD3XH5.

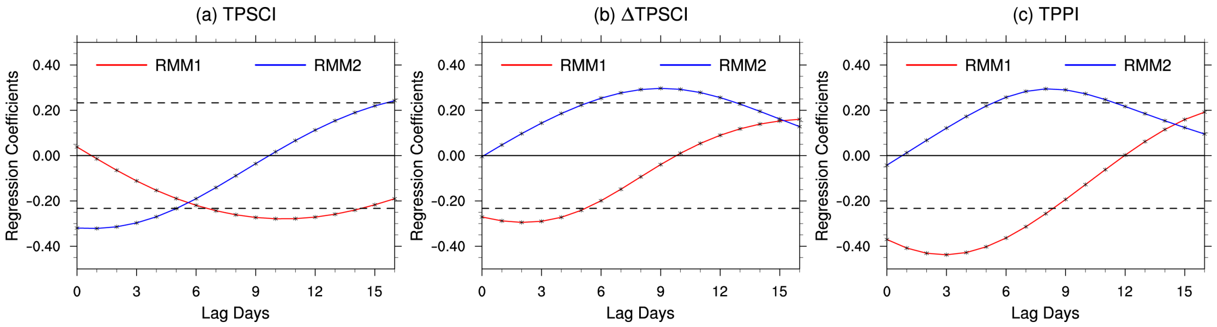

Figure S2. Relationships between the RMM1/RMM2 and indices of snow cover, daily change of snow cover and precipitation over the Tibetan Plateau based on the regression method. Regression of the 20–100-day filtered daily anomalous (a) Tibetan Plateau Snow Cover Index (TPSCI); (b) daily change of the TPSCI; (c) Tibetan Plateau Precipitation Index (TPPI) on to RMM1 (red lines) and RMM2 (blue lines). The three indices are the same as that used in Figure 2 in the manuscript. Lag days indicate that the regression coefficients are calculated from the RMM1/RMM2 and the indices that lagged the RMM1/RMM2. All the indices are standardized. The magnitude represents the anomalies corresponding to one standard deviation of the given RMM1/RMM2. Only strong MJOs were contained for the regression. As the indices are 20–100-day filtered, the effective degrees of freedom (EDoF) would reduce due to high autocorrelation. We calculated the EDoF using the method presented by Davis[S1] and Chen[S2]. The EDoF ranges from 56 to 217 for different lag times and three indices. Using the average value, 122, as the EDoF, the 99 % confidence level (around 0.23) is marked by the dash lines. Figure S2 was generated using NCAR Command Language (NCL) version 6.3.0, an open-source software package that is free to the public and was developed by UCAR/NCAR/CISL/TDD, http://dx.doi.org/10.5065/D6WD3XH5.

The pair of daily all-season real-time multivariate MJO (RMM) indices (RMM1 and RMM2) was obtained based on an empirical orthogonal function (EOF) analysis of the combined fields of equatorially averaged (15°S–15°N) 850- and 200-hPa zonal wind and satellite-observed OLR. The RMM indices are actually the principal component (PC) time series of EOFs 1 and 2. A positive RMM1 phase corresponds to enhanced equatorial convection near the Maritime Continent, while a positive RMM2 phase corresponds to a dipole equatorial convection pattern with enhanced convection over the tropical western Pacific and suppressed convection over the tropical Indian Ocean. More details about the RMM can be referred in Ref. S3.

Both the regression coefficients of RMM1 and RMM2 with respect to the three indices look like a sine wave (Fig. S2). For the ΔTPSCI (Fig. S2b), the RMM1 shows a significant negative correlation with respect to the ΔTPSCI lagged 0–5 days. On the other hand, the RMM2 shows non-significant correlation with the ΔTPSCI. The regression coefficients of RMM1 and RMM2 have a nearly quadrature relationship in lead-lag time, in agreement with the lead-lag correlations between RMM1 and RMM2. As the above mentioned, the positive RMM1 phase corresponds to enhanced equatorial convection near the Maritime Continent, while the negative RMM1 phase corresponds to suppressed tropical convection. The significant negative correlation between RMM1 and the ΔTPSCI at lag = 0 days suggests that the negative anomalous ΔTPSCI corresponds to a positive RMM1 phase (enhanced convection near the Maritime Continent). For the opposite situation, a negative RMM1 phase (suppressed convection near the Maritime Continent) leads to a positive anomalous ΔTPSCI. Moreover, the regression coefficient of the TPSCI lags that of ΔTPSCI by 1/4 cycle (Fig. S2a–b). And the relationship between the ΔTPSCI and the RMM is the same as that between the TPPI and the RMM (Fig. S2b–c).

The above analysis reveals that TPSC increases/decreases when the eastward-propagating MJO suppressed/enhanced convection is located over the Maritime Continent, as well as the precipitation over the Tibetan Plateau increases/decreases. The results is consistent with that revealed by composites methods in the manuscript.

References

S1. Chen, W. Y. Fluctuations in northern hemisphere 700 mb height field associated with the Southern Oscillation. *Mon. Weather. Rev.* **110**, 808–823, doi: 10.1175/1520-0493(1982)110<0808:finhmh>2.0.CO;2 (1982).

S2. Davis, R. E. Predictability of sea-surface temperature and sea-level pressure anomalies over North Pacific Ocean. *J. Phys. Oceanogr.* **6**, 249–266, doi: 10.1175/1520-0485(1976)006<0249:possta>2.0.co;2 (1976).

S3. Wheeler, M. C. & Hendon, H. H. An all-season real-time multivariate MJO index: Development of an index for monitoring and prediction. *Mon. Weather. Rev.* **132**, 1917–1932, doi:10.1175/1520-0493(2004)132<1917:aarmmi>2.0.co;2 (2004).

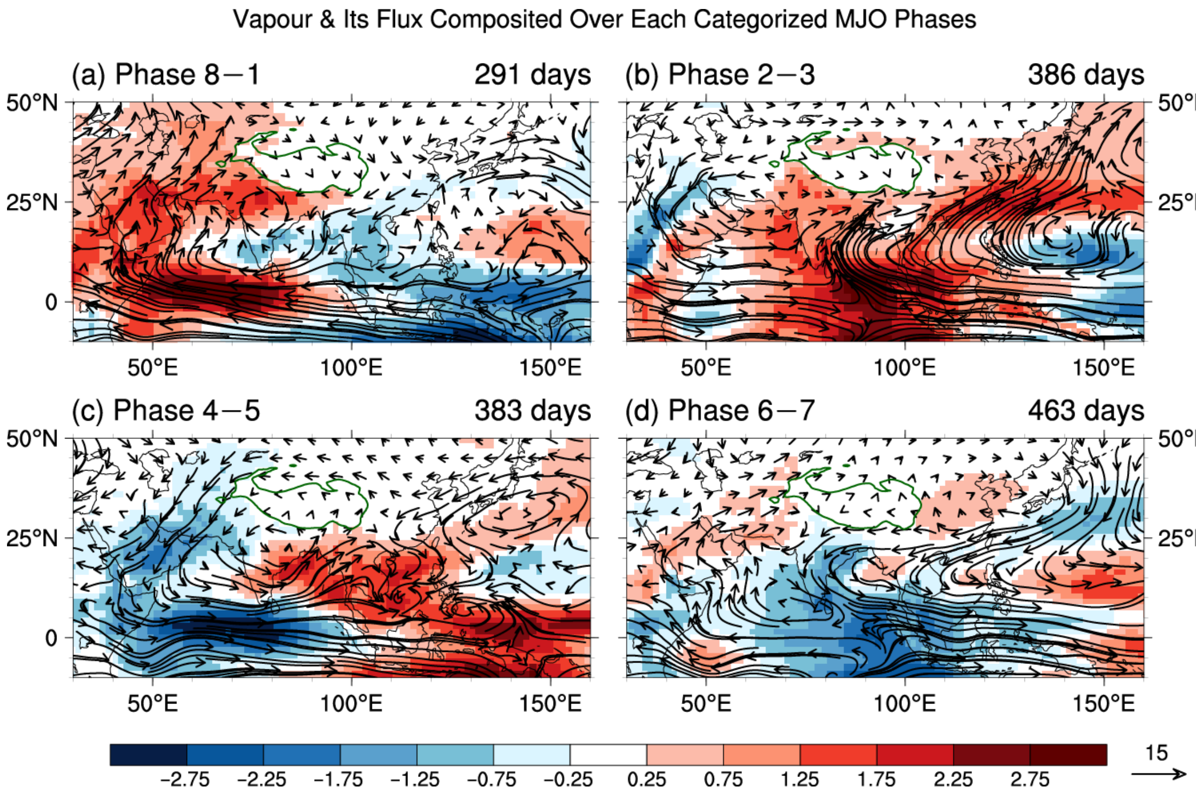

Figure S3. Relationships between the categorized phases of the eastward propagating MJO and the vertical integral of water vapour and its flux. The 20–100-day filtered anomalous vertical integral of water vapour (shadings; unit: kg m^{-2}) and its flux (vectors; unit: kg m^{-1} s^{-1}) for categorized MJO phases is shown. The green contours outline the area 3,000 m above sea level. The reference magnitude used for the flux is 15 kg m^{-1} s^{-1}. Figure S3 was generated using NCAR Command Language (NCL) version 6.3.0, an open-source software package that is free to the public and was developed by UCAR/NCAR/CISL/TDD, http://dx.doi.org/10.5065/D6WD3XH5.

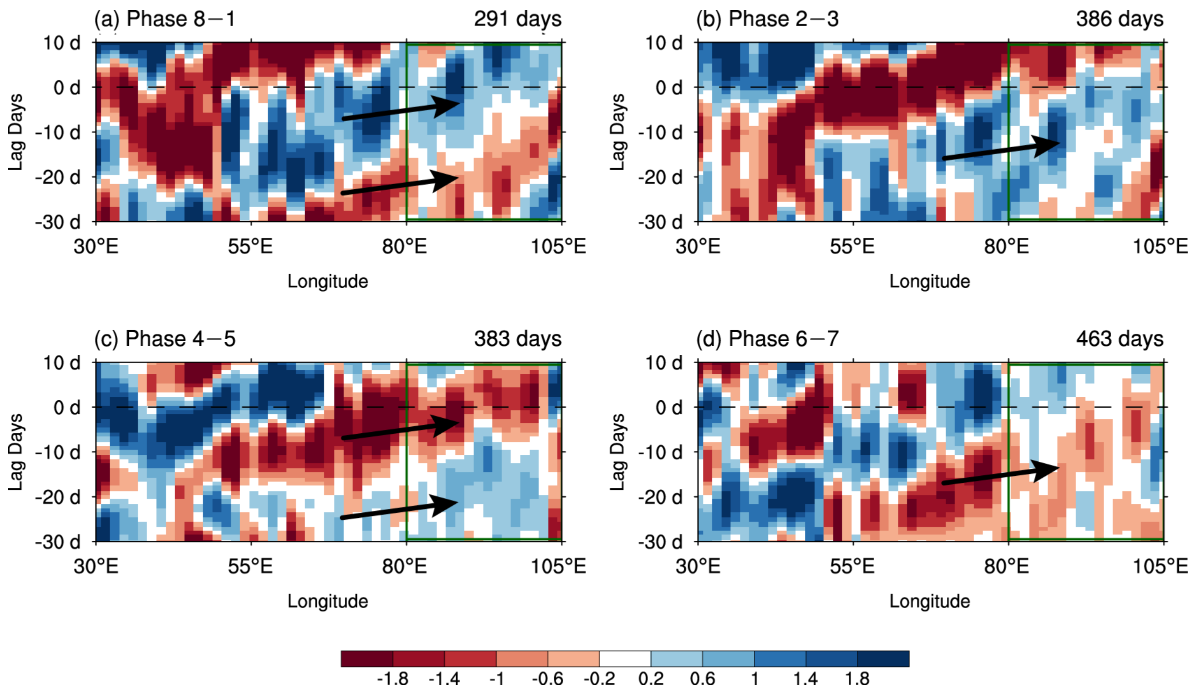

Figure S4. The zonal propagation of the anomalous vertical integral moisture horizontal advection from the area upstream of the Tibetan Plateau. Hovmöller diagrams (time vs. longitude) of 20–100-day filtered daily anomalous vertical integral moisture horizontal advection (unit: 10^{-6} kg m^{-2} s^{-1}) averaged over 27–40°N are shown. The x-axis represents longitude. The y-axis represents the lead-lag time between the anomalies and the categorised phases of the MJO. Negative lag days mean the previous composites in certain categorised phases. The green rectangle shows the TP region. The black arrows represent the propagation of the anomalous advection from the area upstream of the Tibetan Plateau. Figure S4 was generated using NCAR Command Language (NCL) version 6.3.0, an open-source software package that is free to the public and was developed by UCAR/NCAR/CISL/TDD, http://dx.doi.org/10.5065/D6WD3XH5.

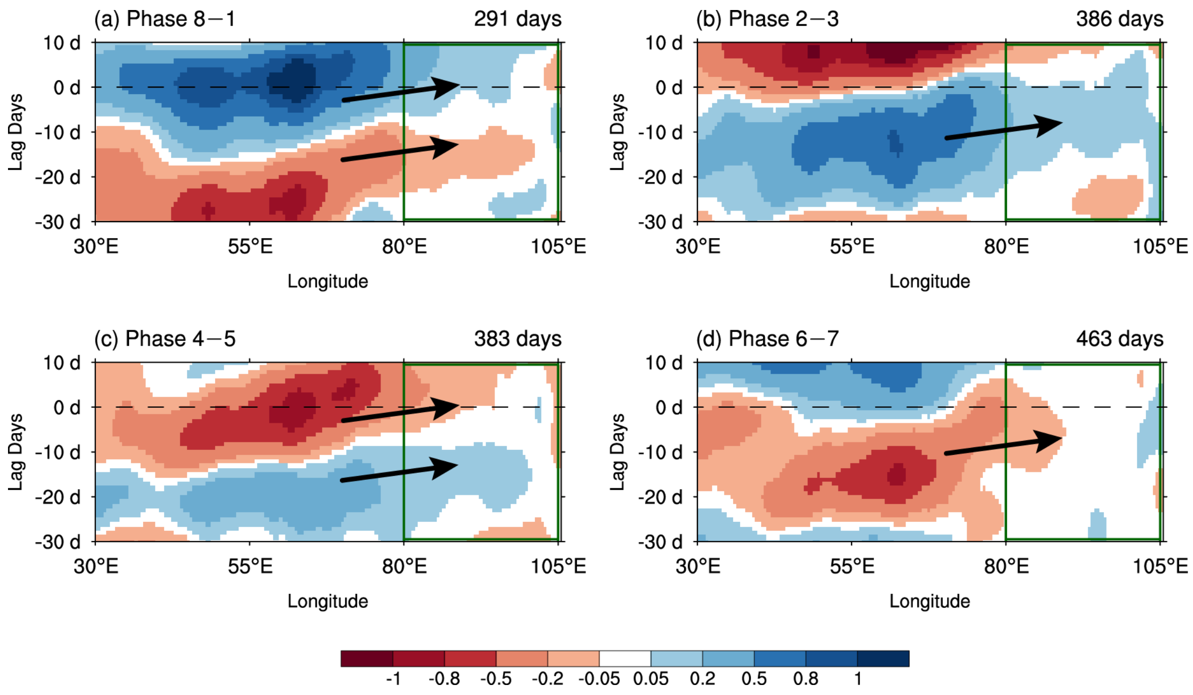

Figure S5. The zonal propagation of the anomalous vertical integral of water vapour from the area upstream of the Tibetan Plateau. As Figure S4, but for the anomalous vertical integral of water vapour. The unit is kg m^{-2}. Figure S5 was generated using NCAR Command Language (NCL) version 6.3.0, an open-source software package that is free to the public and was developed by UCAR/NCAR/CISL/TDD, http://dx.doi.org/10.5065/D6WD3XH5.

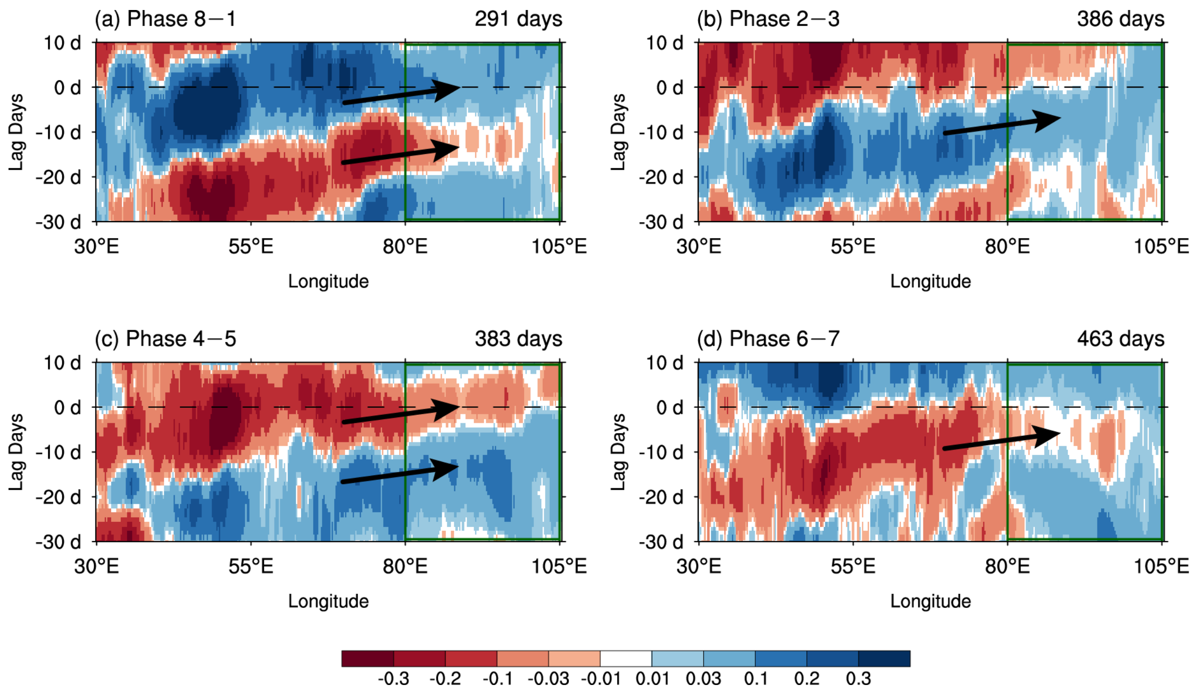

Figure S6. The zonal propagation of the anomalous precipitation from the area upstream of the Tibetan Plateau. As Figure S4, but for the anomalous precipitation. The unit is mm day^{-1}. Figure S6 was generated using NCAR Command Language (NCL) version 6.3.0, an open-source software package that is free to the public and was developed by UCAR/NCAR/CISL/TDD, http://dx.doi.org/10.5065/D6WD3XH5.

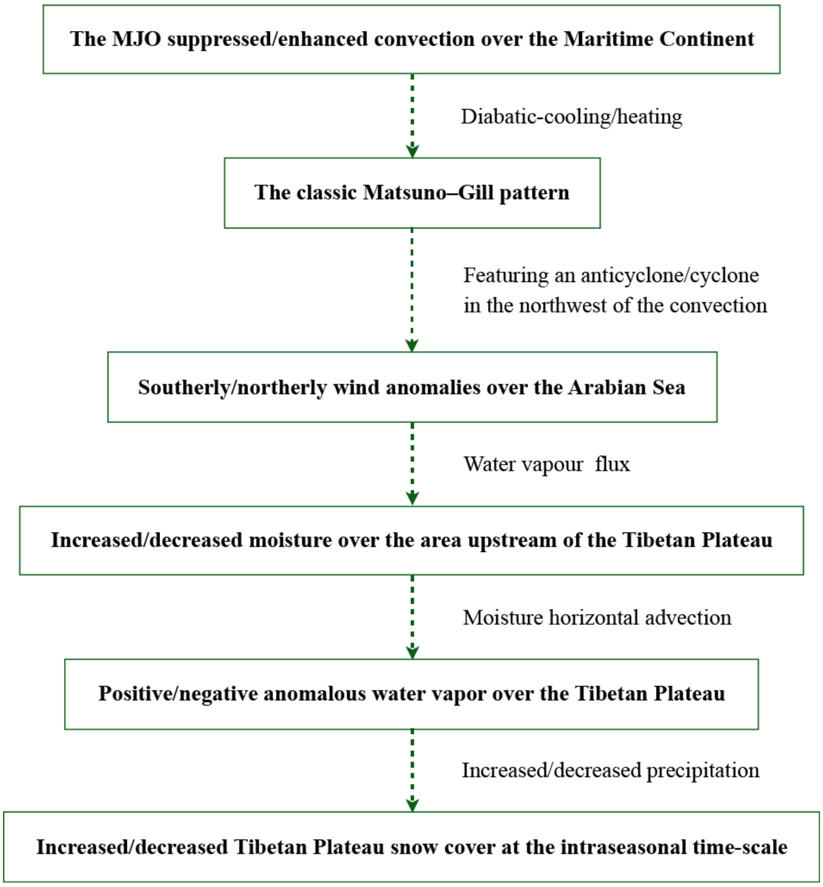

Figure S7. The schematic diagram of the possible mechanism regarding the influence of the MJO on Tibetan Plateau snow cover.
